# TENSIOMETRIC ANALYSIS OF MESHES USED IN ABDOMINAL VENTRAL WALL DEFECTS IN RATS

**DOI:** 10.1590/0102-6720201700030001

**Published:** 2017

**Authors:** Carlos Alberto Lima UTRABO, Nicolau Gregori CZECZKO, Cesar Roberto BUSATO, Mário Rodrigues MONTEMÓR-NETTO, Leandro LIPINSKI, Osvaldo MALAFAIA

**Affiliations:** 1 Postgraduate Program in Principles of Surgery, Evangelic Faculty of Paraná, Curitiba, PR; 2 Medical School, State University of Ponta Grossa, Ponta Grossa, PR, Brazil

**Keywords:** Surgical mesh, Polypropylene, Polyglecaprone, Tensiometry, Abdominal hérnia, Rats

## Abstract

**Background:**

Among the various strategies to avoid exaggerated foreign body reaction in the treatment of hernias is the limitation of the amount of polypropylene or the use of absorbable material.

**Aim:**

To evaluate the healing of defects in the abdominal wall of rats, comparing microporous polypropylene, macroporous polypropylene and polypropylene/polyglecaprone at the 30º, 60º and 120º postoperative day.

**Methods:**

Wistar rats were submitted to defect production in the ventral abdominal wall, with integrity of the parietal peritoneum. Prolene^®^, Ultrapro^®^ and Bard Soft^®^ meshes were used in the correction of the defect. Nine subgroups of 10 animals were submitted to euthanasia at 30^th^, 60^th^ and 120^th^ postoperative day. Fragments of the abdominal wall of the animals were submitted to tensiometric analysis.

**Results:**

The tensiometry at the 30^th^ postoperative day showed greater resistance of the tissues with Bard Soft^®^ (macroporous mesh) in relation to the tissues with Prolene^®^ (microporous mesh). On the 60^th^ postoperative day Bard Soft^®^ maintained the superior resistance to the tissues comparing to Prolene Mesh®. On the 120^th^ postoperative day the tissues repaired with Ultrapro^®^ (macroporous mesh) proved to be more resistant than the ones by Prolene^®^ (microporous mesh) and Bard Soft^®^ (macroporous mesh).

**Conclusion:**

The tissues repaired with macroporous meshes showed greater resistance than with microporous meshes at all stages, and at 120 days postoperative Ultrapro® performed better than the others.

## RESUMO

**Racional:**

Dentre as várias estratégias para evitar exagerada reação de corpo estranho no tratamento das hérnias está a limitação da quantidade de polipropileno na tela ou utilização de material absorvível.

**Objetivo:**

Avaliar a cicatrização de defeito em parede abdominal de ratos, comparando-se as telas de polipropileno microporosa, polipropileno macroporosa e polipropileno/poliglecaprone.

**Métodos:**

Em 90 ratos Wistar foi produzido defeito na parede abdominal ventral, com integridade do peritônio parietal. Na correção foram utilizadas as telas Prolene®, Ultrapro® e Bard Soft®. Nove subgrupos de 10 animais foram submetidos à eutanásia no 30º, 60º e 120º dia do pós-operatório. Fragmentos da parede abdominal dos animais foram submetidos à análise tensiométrica.

**Resultados:**

A tensiometria aos 30dias mostrou maior resistência do tecido com tela Bard Soft^®^ em relação à de Prolene^®^; no 60º dia a Bard Soft^®^ manteve a resistência superior ao Prolene^®^; no 120º dia a reparação com tela macroporosa Ultrapro^®^ mostrou-se mais resistentes que a de Prolene^®^ e Bard Soft^®^.

**Conclusão:**

Os tecidos reparados com telas macroporosas demonstraram maior resistência do que as microporosas em todas as fases, sendo que aos 120 dias de pós-operatório a Ultrapro^®^ teve melhor desempenho que as demais.

## INTRODUCTION

The standard procedure for the surgical correction of the incisional hernia is performed with the use of meshes^3^ ^,^ ^7^ ^,^ ^12^. The most widely used material is polypropylene which causes rapid acute inflammatory response followed by chronic foreign body reaction that persists for months and years after the surgical procedure. Among the various strategies used to avoid exaggerated foreign body reaction is the limitation of the amount of polypropylene or the use of absorbable material that provides initial resistance and is quickly reabsorbed by reducing local inflammation^14^.

Studies focusing on the behavior of macroporous prosthetic material, including polypropylene, have been focused on the geometry and quantity of the implanted material. Attempts to reduce the amount of foreign body focused on the design of the macropores and the absorbable and nonabsorbable components of the meshes. The new designs promoted the development of the classification of the meshes as high, medium and low weight, respectively values ​​above 80 g/m², between 50-80 g/m² or below 50 g/m². Some authors define as ultra light material with a density below 35 g/ m². The pore size represents an important factor for new designs, as well as the filaments themselves and their spatial distribution^6^.

The most important concept in the development of hernia surgery in recent years is the use of low weight meshes with large pores. The new generation has shown advantages in improving postoperative comfort and in chronic postoperative pain. The Ultrapro^®^ represents a new member in the group of low weight with large pores. It consists of a monofilament with low weight and large pores, more than 3 mm in polypropylene, with the addition of a Monocryl® (polyglecrapone 25) absorbable component that optimizes the implant, increasing the resistance of the corrected wall in the first weeks after the repair. Monocryl^®^ is fully absorbed without increased cellularity, inflammatory process and intense fibrotic reaction between 84-140 days. The mesh being partially absorbable and absorbing part of its components, reduces the amount of foreign matter without compromising its biomechanical resistance^10^.

It is known that polypropylene meshes cause early and persistent fibrosis. The reduction is directly proportional to the reduction of its weight and the formation of the fibrosis bridges, inversely proportional to the size of the pores^11^.

The present experiment aimed to study the effect of the application of absorbable and non-absorbable meshes as reinforcement in the closure of preperitoneal abdominal wall lesions in rats.

## METHOD

The project was submitted and approved by the Animal Use Ethics Committee (CEUA) of the State University of Ponta Grossa, Process nº 037/2014.

**FIGURE 1:**
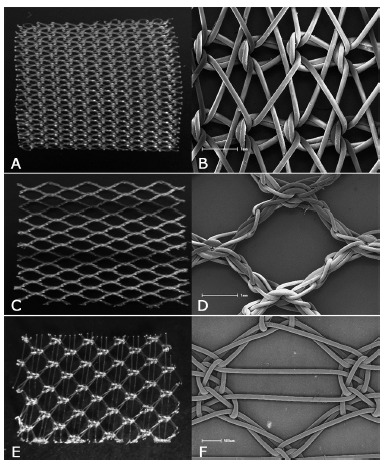
Meshes used in the experiment and, next to it, their scanning electron microscopy visualization: A and B) Prolene®; C and D) Ultrapro®; E and F) Bard Soft®

A total of 90 Wistar rats (*Rattus norvergicus albinus*), males, young adults, with three months weighing 280-300 g from the Central Biotério of the State University of Ponta Grossa were used. The animals were divided into three groups of 30 (Prolene^®^, Ultrapro^®^ and Bard Soft^®^. All groups underwent similar surgical procedures. In the Prolene^®^ group, the non-absorbable, high-density monofilament mesh with micropores measuring approximately 0.9 mm², composed of polypropylene with an estimated weight of 100 g/m^2^ (Figures 1A and 1B) was used. In the Ultrapro^®^ group was used the partially absorbable, low density monofilament with an estimated weight of 28 g/m² with macropores of 3-4 mm in size composed of a combination of equal parts of polypropylene and polyglecaprone (Figures 1C and 1D). In the Bard Soft^®^ group, the non-absorbable, low density monofilament mesh with an estimated weight of 44 g/m² with macropores of approximately 6.29 mm², composed of polypropylene (Figures 1E and 1F), was used

Each group was divided into three subgroups of 10, with G1, G3 and G7 with Prolene^®^ implant being evaluated respectively at 30, 60 and 120 postoperative days; group Ultrapro^®^ G2, G4 and G8, evaluated on the same days; group Bard Soft^®^, G5, G6 and G9 in an identical manner (Table 1).

**TABLE 1:** Distribution of groups and subgroups

Groups	Mesh	Period of time	Subgroups
Prolene	Prolene®	30 days	G1
		60 days	G3
		120 days	G7
Ultrapro	Ultrapro®	30 days	G2
		60 days	G4
		120 days	G8
Bard Soft	Bard Soft®	30 days	G5
		60 days	G6
		120 days	G9

The rats underwent preoperative fasting of 12 h and anesthetized with atropine sulfate (0.05 mg/kg body weight) intraperitoneally, and after 10 min the mixture of 2% xylazine hydrochloride (10 mg/kg) and hydrochloride of ketamine 10% (25 mg/kg). When necessary, half the dose was repeated after 20-30 min. They were submitted to postoperative analgesia with oral acetaminophen in the dose of 40 drops for each 500 ml of water offered in the first two days.

**FIGURE 2:**
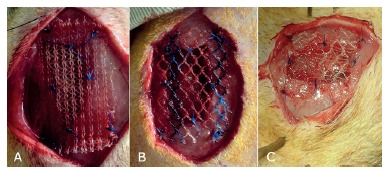
Meshes applied to the abdominal wall defect: A) Prolene^®^; B) Ultrapro^®^; C) Bard Soft^®^

A defect of 1x2 cm was produced in the abdominal wall, preserving the integrity of the parietal peritoneum. Correction was performed using each of the 1.5x2.5 cm area meshes fixed in the extraperitoneal position through four separate stitches of Prolene^®^ 5-0 wire securing the mesh angles to the aponeurosis of the abdominal wall, 0.5 cm from the edge of the lesion, and four separate stitches interspersed with the first ones, securing the lesion at the edges of the lesion (Figure 2 - A, B and C). The skin was sutured with intradermal 5-0 mononylon intradermal.

Euthanasia was performed at the defined dates (Table 1). At that time, macroscopic evaluation of the operative wound and the peritoneal cavity was made, and a fragment of the wall was removed, which, divided into a median section, gave rise to a cranial and a caudal fragment. The segment containing the mesh and musculature (cranial), without the skin, was submitted to tensiometric tests. The caudal fragments were maintained in 10% formalin solution. The cranial segments were placed in vials with isotonic saline solution and kept in vials with ice.

For tensiometry, the Shimadzu (Japan) model AG-I tensiometer was used with Trapezium 2 software, where the data provided for the test (area and thickness of the tissue) and the results obtained were recorded. The tests were performed at a temperature of 24º C. The apparatus was calibrated for a speed of 50 mm/min. The results were expressed as N/cm. The cranial fragment was attached to the tensiometer by the muscular tissues next to the suture site.

### Statistical analysis

The means between the results of the tensiometry were submitted and approved in the normality test of KS (Kolmogorov and Smirnov), suggesting parametric inference tests. From unpaired ANOVA, evidence of significant differences, at the 5% level, was found between treatments in relation to tensiometry in the replicates, thus rejecting the null hypothesis at these points of observation.

## RESULTS

### Macroscopic and tensiometric evaluations

No animal presented any complications, and the edges of the mesh fixation to wounds were fully coapted in all animals.

In tissue tensiometry with implanted tissue, it was found that the rupture always occurred outside the suture line of the mesh in the abdominal wall.

The mean stress at break of subgroup G5 (Bard Soft^**®**^ ) at 30 days was 32.32 N/ cm, higher than the mean tension at subgroup G1 (Prolene^**®**^ ) of 21.73 N/cm (p<0.01). The G2 (Ultrapro^**®**^ ) subgroup with mean tension of 24.49 N/cm at 30 days did not present a statistically significant difference in relation to G5 (Bard Soft^**®**^ ) subgroups with mean rupture stress of 32.32 N/cm, and G1 (Prolene^**®**^ ) with mean tension at the same time of 21.73 N/cm (p>0.05).

On the 60^th^ postoperative day, the subgroup G6 (Bard Soft^**®**^ ) showed a mean rupture tension of 36.36 N/cm statistically superior to the subgroup G3 (Prolene^**®**^ ), with a mean tension of rupture of 24 N/cm (p<0.05). The comparison between G4 (Ultrapro^**®**^ ) subgroup that presented mean tension at 26.12 N/cm at 60 postoperative days, G3 (Prolene^**®**^ ) subgroups with mean tension at 24 N/cm and G6 (Bard Soft^**®**^ ) with mean rupture stress of 36.26 N/cm, did not show a statistically significant difference (p>0.05).

**TABLE 2:** Mean and standard deviation of burst voltage (N/cm)

Treatment	Repetitions			
	Time (days)			
	30	60	120	
Materials	Prolene	21.73±4.47	24.00±11.36	33.40±9.99
	Ultrapro	24.49±7.78	26.12±7.75	46.77±6.54
	Bard Soft	32.32±8.47	36.26±7.41	37.78±5.90

**TABLE 3:** Comparison of the means of rupture stress (N/cm)

Period of observation (days)	Mean of tensiometry	p	
30	G5>G1	p<0.01	p=0.0073
	G2=G5	p>0.05 (ns)	
	G2=G1	p>0.05 (ns)	
60	G6>G3	p<0.05	p=0.0117
	G4=G3	p>0.05 (ns)	
	G4=G6	p>0.05 (ns)	
120	G7=G9	p>0.05 (ns)	p=0.0020
	G8>G7	p<0.01	
	G8>G9	p<0.05	

**FIGURE 3:**
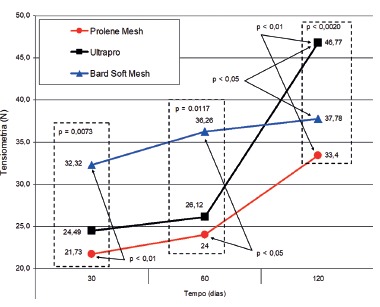
Comparison of the mean tensile stress

On the 120^th^ postoperative day, the tensiometric analysis showed a statistically significant difference between the subgroup G8 (Ultrapro^**®**^ ), which presented a mean tension at 46.77 N/cm, higher than the tension of the subgroups G7 (Prolene^**®**^ ) with mean tension of rupture of 33.4 N/cm (p<0.01) and subgroup G9 (Bard Soft^**®**^ ) with mean rupture stress of 37.78 N/cm. Among the G7 and G9 subgroups there was no statistically significant difference (p>0.05) (Tables 2 and 3, Figure 3). 

## DISCUSSION

### Surgical meshes

The evolution of polypropylene prostheses has revolutionized surgery to correct abdominal wall defects. The creation of the low-weight prosthesis theoretically induced a smaller foreign body reaction, resulting in an improvement of the abdominal wall, causing less contraction of the mesh and providing better incorporation of the abdominal wall^5^ ^,^ ^9^ ^,^ ^4^.

Numerous modifications in the designs of the prostheses have been investigated for the reduction of the complications related to the healing process. Alteration of the architecture, increase of the pore area and low weight mesh, are the most important predictors of the biocompatibility performance of synthetic meshes. Those with broad pores show less inflammatory infiltrate, connective tissue and fibrosis bridges^18^.

The prostheses for this study were chosen based on the wide use of polypropylene today, and considering that there are few studies comparing the low and high density meshes used in the extraperitoneal space in the period over 100 days.

Greca et al.^5^ in studies with dogs comparing low and high-weight polypropylene meshes on the abdominal wall including the peritoneum had incidence of 20% of seroma in both, 5% of infection in high-weight Prolene^**®**^ , dehiscence in 9.1% in the low-weight group and 4.6% in the high-weight group, and there was no incorporation of the mesh in 5% of the high-weight group. In this study, there was no complication in the group as a whole.

In tissue tensiometry rupture was observed always outside the suture line, a result also obtained by Aydos et al.^1^ and Pundek et al.^15^

Cobb et al.^4^ showed that after the implant of high-weight and low-weight mesh showed a significant increase of deposition of collagen type I on the one with larger pores of low-weight. The rupture tension increased after 30 days of implantation in dogs, although the same occurred in the high-weight mesh with smaller pores. A similar study evaluated the high-weight polypropylene mesh with pores smaller than 1 mm with a low-weight and pores larger than 3 mm (Vypro^®^), but with an absorbable component. The wide pore mesh was integrated with loose deposition of fibrosis interspersed with fatty tissue. In contrast, that of pores smaller than 1 mm, was incorporated entirely with peri filamentary granulomas and scar tissue, forming bridges between the pores. It has been proven that the great distance between filaments avoids the formation of these bridges.

The results of this study, in the comparison of the mean of rupture tension between the G1 (Prolene^®^) and G5 (Bard Soft^®^) subgroups on the 30^th^ day and between the G3 (Prolene^®^) and G6 (Bard Soft^®^) subgroups on day 60 showed greater resistance of the subgroups G5 and G6 (Bard Soft^®^), and on the 120^th^ day a significant increase of resistance in subgroup G8 (Ultrapro^®^) in relation to subgroups G7 (Prolene^®^) and G9 (Bard Soft^®^). The pore size of the mesh has important influence on the biocompatibility of the foreign body after the implant.

The results of this study show that despite fixation of the prosthesis with only four separated stitches in the aponeurosis and four intercalated fixation of the prosthesis in the border of the lesion, the incorporation was sufficient to avoid the suture dehiscence and the rupture of the suture line during the tensiometry in all the prostheses used^16^.

The results of this study also showed that at 120 days there is greater resistance of the corrected wall with the Ultrapro^®^ prosthesis, which has pores with a diameter greater than 3 mm² and a weight of approximately 28 g/m², compared to Prolene^®^ prostheses - whose pores have a diameter of less than 1 mm² and approximate weight of 100 g/m² - and Bard Soft^®^ - with a pore size of approximately 6.29 mm², but weighing approximately 44 g/m². This increase in resistance is shown to be even higher when tissue resistance is compared with the implantation of the Bard Soft^®^ prosthesis in 30 and 60 days, which has a larger pore diameter^5^ ^,^ ^1^ ^,^ ^13^. It is observed that the Bard Soft^®^ mesh despite having a pore with a larger diameter than the Ultrapro^®^, has a higher density depending on the mesh design^2^.

White et al.^17^ reported that complete tissue incorporation into the recipient tissue is an important requirement for obtaining solid repair. The degree of infiltration of the receptor tissue with the biomaterial depends on the pore size. The incorporation of the prosthesis into the recipient tissue is proportional to the degree of its porosity. The infiltration of fibrocytes and collagen from the recipient tissue into the prosthesis with adequate porosity occurs in approximately one month. Adequate incorporation requires pores between 75 and 100 μm in size. The mesh with polypropylene monofilament, with pore greater than 100 μm, produces complete infiltration of the receptor tissue incorporating the entire prosthesis.

Studies^4,13,8^ confirm good integration of high- and low-weight prostheses in the repaired tissues. There is greater encapsulation in the high-weight meshes and consequent hardening of the corrected wall and better distribution of the of fibrosis between the filaments of the low-weight, providing better elasticity and malleability of the corrected wall.

## CONCLUSIONS

The tissues repaired with macroporous meshes showed greater resistance than the microporous ones in all the phases, being that at 120 days of postoperative the Ultrapro^®^ had better performance than the others.
